# Oncolytic HSV1716 attenuates tumor-killing activity of infected primary NK cells

**DOI:** 10.3389/fimmu.2026.1895832

**Published:** 2026-08-03

**Authors:** Katharina H. Susek, Dani Holla, Claire Marsal, Muhammad Kashif, Jonathan Albert, Viktoria Flore, Mari Gilljam, Maria Karvouni, Stephan Meinke, Arnika K. Wagner, Colin Powers, Robert Allen, Evren Alici

**Affiliations:** 1 Center for Hematology and Regenerative Medicine, Department of Medicine Huddinge, Karolinska Institute, Stockholm, Sweden; 2 Sorrento Therapeutics, San Diego, CA, United States

**Keywords:** cancer immunotherapy, virus-carrying NK cells, HSV1716, natural killer cell, oncolytic virus

## Abstract

Oncolytic herpes simplex virus type-1 (HSV1)–based therapies engage innate immune responses, including natural killer (NK) cells, which are regarded as antiviral effector cells that eliminate virus-infected tumor targets. In this study, we examined interactions between the HSV1–derived oncolytic virus HSV1716 and primary human NK cells. Co-culture experiments revealed increased activation and degranulation of NK cells in response to HSV1716-infected tumor cells, despite the downregulation of ligands for NK-cell activating receptors on infected targets. Following co-culture with infected tumor cells, but not after incubation with viral inoculum alone, viral gene expression and increased viral copy numbers were detected in NK cells, indicating enhanced viral acquisition and persistence associated with target-cell contact. HSV1716-infected NK cells displayed impaired tumor cell killing ability. Single-cell sequencing analysis revealed downregulation of key NK effector genes alongside alterations in stress-response pathways in HSV1716 infected NK cells. Together, these findings demonstrate that primary human NK cells are infected by HSV1716 and undergo functional and phenotypic changes, leading to a diminished cytotoxic capacity. Given the emerging role of NK cell–based therapies in cancer, these findings may be relevant for the design and timing of oncolytic virus–based strategies in the future.

## 1 Introduction

Oncolytic viruses (OV) are naturally occurring or genetically engineered viruses that selectively infect and kill malignant cells while simultaneously promoting antitumor immune responses. Over the past decade, OV have advanced through early- and late-phase clinical trials, positioning oncolytic virotherapy as a distinct immunotherapeutic modality. Among the most clinically advanced OV platforms is herpes simplex virus (HSV1). Talimogene laherparepvec (T-VEC), an HSV-based OV encoding granulocyte–macrophage colony-stimulating factor (GM-CSF), was approved by the U.S. Food and Drug Administration in 2015 for the treatment of unresectable melanoma (1). More recently, the triple-mutated HSV OV G47Δ (teserpaturev, Delytact^®^) demonstrated clinical benefit in recurrent glioblastoma and received conditional, time-limited approval in Japan in 2021 following a pivotal phase II study (2). Despite these regulatory milestones, the overall clinical efficacy of OV monotherapy remains limited. Both T-VEC and G47Δ are administered intratumorally, restricting their use to accessible lesions and limiting systemic antitumor immunity. Additional challenges include rapid antiviral immune responses, pre-existing HSV immunity, and the immunosuppressive tumor microenvironment. Nevertheless, HSV OV are highly attractive therapeutic platforms due to their large genomic capacity, genetic stability, and amenability to engineering, supporting their continued development in combination therapies and immunomodulatory strategies.

Natural killer (NK) cells are innate lymphocytes that eliminate infected, stressed, or malignant cells without prior antigen sensitization. NK cell activity is dynamically regulated by the balance between activating and inhibitory receptors (3, 4). Key activating receptors include CD16, which mediates antibody-dependent cellular cytotoxicity (ADCC), the natural cytotoxicity receptors NKp30, NKp44, and NKp46, as well as DNAM1 and NKG2D, which recognize stress-induced ligands such as CD112, CD155, and MICA/B. Inhibitory signaling is mediated primarily through killer immunoglobulin-like receptors (KIRs), NKG2A, and TIGIT, which engage HLA class I molecules and related ligands.

NK cells play a critical role in host defense against HSV infection. Individuals with severe or recurrent HSV disease display reduced frequencies of CD56^dim^ CD16^bright^ NK cells and impaired expression of activating receptors, highlighting the importance of NK cell–mediated antiviral responses (5). In the context of oncolytic virotherapy, NK cells readily recognize and kill HSV OV-infected tumor cells. This has been demonstrated in hematological malignancies, where NK cells enhanced cytotoxicity against HSV OV-treated plasma cells (6). However, NK cell antiviral activity can also limit OV persistence and therapeutic efficacy. In glioma models, depletion or functional inhibition of NK cells enhanced the efficacy of HSV OV, indicating that premature clearance of infected tumor cells can impair viral oncolysis and antitumor immunity (7).

These findings suggest that the interaction between NK cells and HSV OV-infected tumor cells is complex and likely regulated in a time-dependent manner, balancing antiviral defense with antitumor efficacy (8). A detailed understanding of these interactions is essential for optimizing HSV OV-based combination strategies. Here, we investigate the phenotypic and functional responses of primary human NK cells upon encountering HSV OV-infected target cells, aiming to elucidate the role of NK cells in HSV OV-mediated immunity.

## 2 Methods

### 2.1 Cell lines and culture

All cell lines were obtained from ATCC. The renal cell carcinoma cell line 786-O wild type (WT) (ATCC^®^ CRL-1932™) and a genetically modified 786-O line expressing the red fluorescent protein mKate2ps were cultured in RPMI-1640 medium supplemented with GlutaMAX (Gibco) and 10% heat-inactivated fetal bovine serum (FBS) (Gibco). The ovarian cancer cell line SKOV3 (ATCC^®^ HTB-77™) was maintained in McCoy’s medium, supplemented with 10% FBS (Gibco). Cells were passaged every 2–3 days using Dulbecco’s phosphate-buffered saline (PBS) (Gibco) and 0.5% trypsin-EDTA (Gibco) and seeded at densities of 6,500 or 3,500 cells/cm², respectively. The multiple myeloma cell lines RPMI8226 (ATCC^®^ CCL-155™) and MM1S (ATCC^®^ CRL-2974™) were cultured in RPMI-1640 medium supplemented with GlutaMAX and 10% non-heat-inactivated FBS and passaged every 2–3 days at a density of 0.3 × 10^6^ cells/mL.

### 2.2 Virus

HSV1716 (Seprehvir), carrying deletions of both ICP34.5-encoding RL1 loci, was kindly provided by Sorrento Therapeutics (9). The HSV1716-dsRed variant expresses dsRed under the CMV promoter.

### 2.3 PBMC and NK cell isolation

This study involved the use of peripheral blood mononuclear cells (PBMCs) obtained as buffy coats from healthy donors through the Karolinska University Hospital Blood Bank under ethical approval 2021-05202. PBMCs were isolated from buffy coats using LymphoPrep™ (Fresenius Kabi) according to the manufacturer’s instructions. NK cells were isolated by negative selection using magnetic beads (Miltenyi Biotec). NK cells were cultured in SCGM medium (CellGenix) supplemented with 10% human serum, IL-21 (20 ng/mL, ImmunoTools) on day 0, and IL-2 (1,000 U/mL, R&D Systems) added daily. NK cells were expanded for 4–5 days prior to functional assays.

### 2.4 Infection of tumor cell lines

Tumor cell lines were resuspended as single-cell suspensions at 1 × 10^6^ cells/mL and infected with HSV1716-dsRed at a multiplicity of infection (MOI) of 1 or mock-infected (MOI 0). Infections were carried out for 1 hour at 37 °C and 5% CO_2_, with brief vortexing every 20 minutes. Following infection, cell suspensions were diluted to 0.5 × 10^6^ cells/mL. RPMI8226 cells were maintained in T25 or T75 flasks until co-culture, whereas 786-O cells were seeded into appropriate flat-bottom culture vessels at a density of 1.56 × 10^5^ cells/cm².

### 2.5 Co-culture experiments

Tumor cells were infected and plated as described above. RPMI8226 cells were resuspended following overnight incubation, counted, and seeded at 150,000 cells/well in 96-well U-bottom plates (Corning). 786-O cells were plated on the day of infection at 50,000 cells/well in 96-well flat-bottom plates (Corning) and incubated overnight. Culture supernatants from infected and non-infected wells were collected, centrifuged at 300 × g, and filtered through 0.45 µm sterile filters (TRP) to remove residual cells. Filtered supernatants were transferred to fresh wells as conditioned medium (CM) prior to NK-cell addition. Target cells were washed twice with DPBS before co-culture. NK cells were added at effector-to-target ratios of 1:1 for RPMI8226 and 3:1 for 786-O, unless indicated otherwise. IL-2 was omitted on the day of co-culture initiation. Final culture conditions were maintained at a 1:1 ratio of effector-cell and target-cell media. Co-cultures were maintained for the indicated time points, and downstream analyses were performed by flow cytometry, qPCR, or Incucyte imaging flow cytometry, qPCR, live cell imaging or single cell sequencing.

### 2.6 Flow cytometry

Antibodies used for flow cytometry are listed in **Supplementary Table 1** (**Supplementary Table 1**) and were titrated prior to use. Cells were stained with Live/Dead dye (Thermo Fisher Scientific), followed by surface staining in PBS containing 2% FBS. For intracellular staining, cells were fixed and permeabilized using Cytofix/Cytoperm (BD Biosciences) according to the manufacturer’s recommendation. Data were acquired on a Beckman Coulter CytoFLEX or BD Symphony flow cytometer and analyzed using FlowJo v10. Gates were set using fluorescence-minus-one or negative controls.

### 2.7 NK cell degranulation assay

Target cells were infected with HSV1716 at MOI 0 or MOI 1 as described above. Adherent targets were seeded at 50,000 cells/well in 96-well flat-bottom plates, while suspension targets were seeded at 150,000 cells/well in round-bottom plates. NK cells were added at a 1:1 effector-to-target ratio and co-cultured for 4 hours in the presence of anti-CD107a antibody (BioLegend, H4A3). PMA/ionomycin stimulation (0.5 ng/mL, Sigma-Aldrich) served as a positive control. Monensin (GolgiStop, BD Biosciences) was added after 1 hour. Cells were subsequently stained for surface markers and intracellular IFN-γ and TNF (BD Biosciences) prior to flow cytometric analysis.

### 2.8 Real-time PCR

Samples for qPCR were generated from co-cultures as described above. NK cells co-cultured with 786-O cells were enriched using CD56 microbeads (Miltenyi Biotec) and isolated by magnetic separation on MS columns. Cells were washed with DPBS and total DNA was extracted using the DNeasy Blood & Tissue Kit (Qiagen). HSV1716 DNA was detected using an HSV1/2 PCR kit targeting the conserved glycoprotein B gene (GeneProof). DNA concentration was measured by NanoDrop, and up to 200 ng DNA was used per reaction. qPCR was performed on a CFX96 real-time PCR system (Bio-Rad).

### 2.9 Live cell imaging

To assess differences in cytotoxic capacity between HSV1716-infected and non-infected NK cells, purified NK cells were co-cultured with 786-O cells expressing mKate2ps, and tumor viability was quantified by loss of red fluorescent signal. 786-O mKate2ps cells were seeded one day prior to NK-cell addition at 2,500 cells/well in 96-well flat-bottom plates. NK cells were generated by co-culture as described above, purified by CD56 microbead selection, and added at effector-to-target (E:T) ratios of 3:1, 1:1, and 1:3. To control for dsRed signal interference, 786-O WT cells were co-cultured with HSV1716-infected NK cells. Tumor viability was monitored by live-cell imaging using an Incucyte S3 system (Sartorius) with a 10× objective (four images/well). NK cells were excluded based on size in the brightfield channel, and dsRed signal was excluded using intensity and size filtering. Tumor viability was quantified as total red integrated intensity multiplied by total brightfield tumor area per image (GCU × µm²). Images were acquired every 30 minutes or 2 hours for up to 48 hours, and values were normalized to the 0-hour time point.

### 2.10 Single-cell RNA sequencing

Cells from co-cultures with 786-O target cells were harvested, washed, filtered through a 40 µm strainer, and sorted by FACS based on CD56 expression alone (non-infected) or CD56 and dsRed expression (infected). As a control, unstimulated NK cells were used. Sorted cells were processed for single-cell RNA sequencing using the Chromium X platform and the GEM-X Universal 3′ Gene Expression v4 kit (10x Genomics) according to the manufacturer’s protocol. Libraries were sequenced on an Illumina platform. Raw single cell sequencing reads were demultiplexed and aligned to the reference human genome GRCh38 using Cell Ranger version 9.0.0. Resulting single-cell gene expression UMI count matrices were imported to programming language R version 4.5.2 using Seurat package version 5.4.0. Cells were retained for downstream analyses if they met the following criteria: cells with fewer than 500 or greater than 6,500 detected genes, fewer than 1000 or more than 25,000 UMIs, or more than 10% mitochondrial transcripts were excluded. Following QC filtering, datasets were normalized by variance-stabilizing transformation with Seurat SCTransform function while regressing out mitochondrial transcript content (10). We used Seurat FindVariableFeatures function to identify highly variable genes using the VST method and selected the top 2,500 variable features. Principal component analysis (PCA) was performed to reduce dimensionality. In order to correct for donor specific batch effects, Harmony integration was applied with a theta parameter of 3. UMAP was generated based on the first 20 Harmony dimensions. A shared nearest-neighbor (SNN) graph was constructed using the Harmony reduction, considering the first 20 dimensions, followed by graph-based clustering using the Louvain algorithm. Clusters were identified using a resolution parameter of 0.35. For comparative analysis between samples, average expression of gene signatures were calculated using Seurat’s AverageExpression function and visualized using heatmaps to compare functional states across samples. Differential gene expression (DGE) analysis was performed using a pseudobulk approach. Gene counts were aggregated across cells from the same biological replicate using Seurat’s AggregateExpression function, and only co-culture samples were included in the comparison of infected versus uninfected conditions. DGE analysis was performed with the edgeR package, using a generalized linear model that included donor and infection status as covariates. Lowly expressed genes were filtered using filterByExpr, library sizes were normalized with calcNormFactors, dispersions were estimated using estimateDisp, and differential expression was assessed using quasi-likelihood F-tests (glmQLFit and glmQLFTest). Genes with a false discovery rate (FDR) < 0.05 and an absolute log_2_ fold change > 1 were considered differentially expressed. The functional enrichment analysis of DGEs was performed using the clusterProfiler package and Kyoto Encyclopedia of Genes and Genomes (KEGG) pathway enrichment was performed with enrichKEGG function while pathways with *P* < 0.05 were considered significantly enriched. The raw sequencing data have been deposited in the Gene Expression Omnibus (GEO) and are available under accession number GSE338854.

### 2.11 Statistical analysis

Statistical analyses were performed using GraphPad Prism v9. Comparisons between two groups were performed using Student’s t-test, and multi-group comparisons were analyzed by two-way ANOVA. P < 0.05 was considered statistically significant. Error bars are shown as mean +/- SD.

## 3 Results

### 3.1 HSV1716 induces oncolysis of tumor cells and modulates ligands for activating and inhibitory NK cell receptors

**Figure 1:**
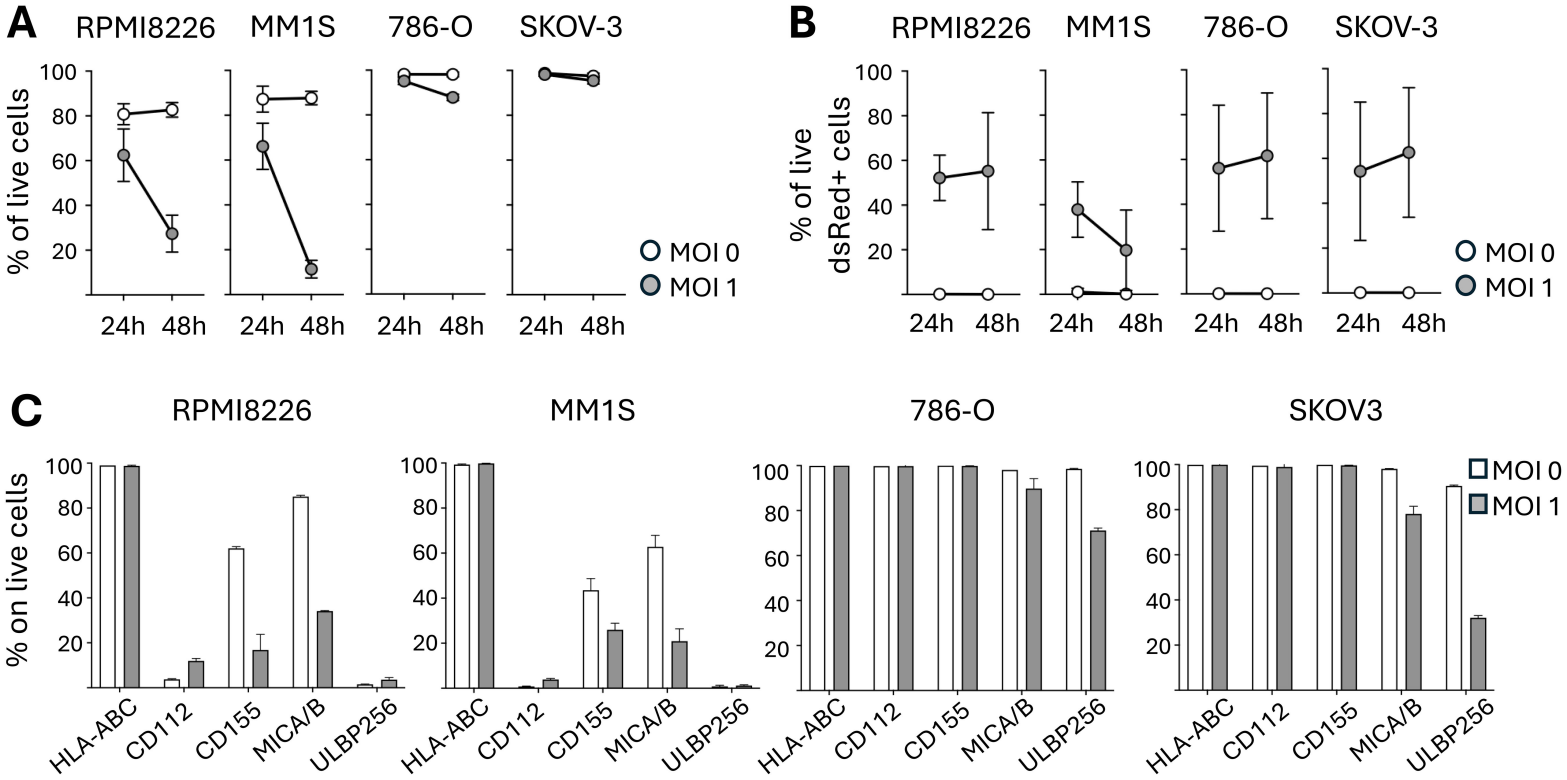
HSV1716 induces oncolysis of tumor cells and modulates ligands for activating and inhibitory NK cell receptors. The human tumor cell lines RPMI8226, MM1S, 786-O and SKOV3 were mock-infected (MOI 0) or infected with HSV1716 expressing dsRed at a MOI of 1. **(A)** Percentage of live tumor cells was assessed at 24 h and 48 h post-infection. **(B)** Percentage of HSV1716-infected target cells, defined as dsRed^+^ cells, was measured at 24 h and 48 h post-infection. **(C)** Expression of ligands for activating and inhibitory NK cell receptors was analyzed by flow cytometry on live cells 24 h post-infection. Each data point represents the mean ± SD from three independent experiments, performed in duplicate.

The ability of HSV1716 to infect different human cancer cell lines was assessed with HSV1716-dsRed, expressing the red fluorescent protein dsRed under the CMV promoter. Infection kinetics were assessed using HSV1716-dsRed at MOI 1 over 48 hours (**Figures 1A, B**). Susceptibility to infection and lysis varied by cell line. The multiple myeloma lines RPMI8226 and MM1S exhibited faster viability loss and higher dsRed and gE expression than the solid cancer lines 786-O and SKOV3. To correlate these phenotypic changes with NK cell cytotoxicity, we analyzed the expression of activating ligands 24 hours post-infection (**Figure 1C**). While HLA-A/B/C levels remained stable, other ligands were significantly modulated. CD112, which serves as both an HSV entry receptor and a ligand for DNAM1/TIGIT, slightly increased on RPMI8226 and MM1S and remained unchanged on 786-O and SKOV3. CD155 expression was reduced in both MM cell lines. Additionally, MICA/B was downregulated across all tested lines, while ULBP decreased on 786-O and SKOV3. These results demonstrate that HSV1716-mediated oncolysis is accompanied by a significant shift in the surface expression of ligands for key NK cell activating receptors.

### 3.2 NK cells increase degranulation in response to HSV1716-infected target cells

**Figure 2:**
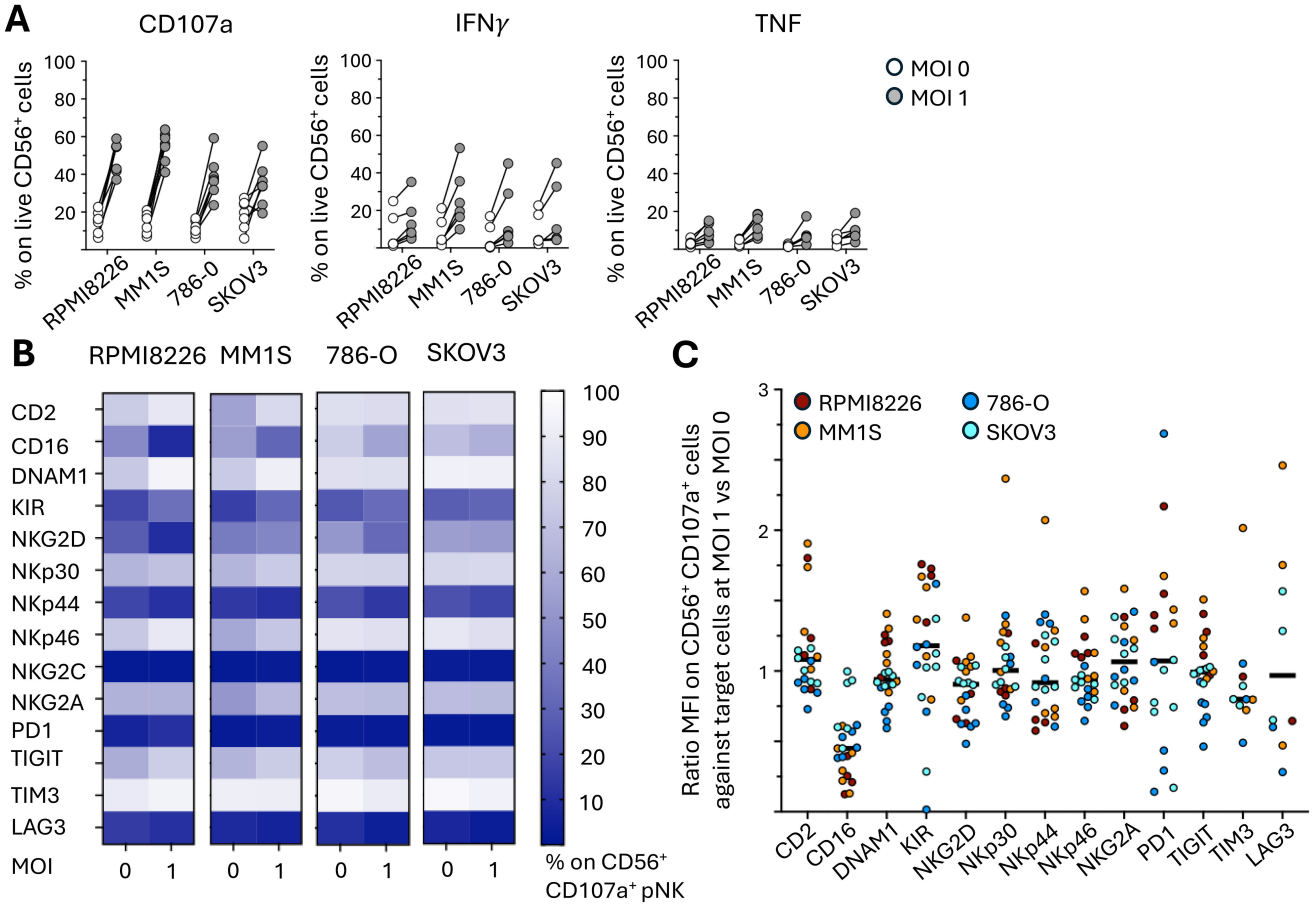
NK cells increase degranulation in response to HSV1716-infected target cells. Target cells were mock-infected (MOI 0) or infected with HSV1716 expressing dsRed at an MOI of 1, 24 h prior to initiation of the degranulation assay. **(A)** Expression of CD107a, IFN-γ, and TNF was assessed on live CD56^+^ NK cells. Each dot represents the mean value from one donor, with experiments performed in duplicate. **(B)** Expression of activating and inhibitory receptors was analyzed on live CD56^+^ CD107a^+^ NK cells. NK cells from 5–6 different donors were analyzed, with experiments performed in duplicate. **(C)** Ratio of mean fluorescence intensity (MFI) on CD56^+^ CD107a^+^ degranulating against infected (MOI 1) versus non-infected (MOI 0) target cells. Each dot represents the ratio of one donor against one target cell line.

To evaluate infection-induced cytotoxicity, we measured CD107a degranulation as well as IFN*γ* and TNF secretion in NK cells after a four-hour co-culture with HSV1716-infected or mock-infected target cells (**Figure 2A**). Degranulation increased approximately three-fold against infected cells, while cytokine secretion doubled. Phenotypic analysis of degranulating NK cells revealed a distinct surface receptor profile on degranulating CD107a^+^ NK cells (**Figures 2B, C**). We observed a significant activation-induced decrease in CD16 expression, accompanied by modest downregulation of TIM3 and increase in KIR expression on NK cells degranulating against all target cells. Other receptor changes were more target cell-line specific. The expression of DNAM1, NKp46 and TIGIT increased following contact with the infected MM cell lines RPMI8226 and MM1S. However, DNAM1 and NKG2D expression decreased following contact with infected 786-O cells. Collectively, these results indicate that NK cells exhibit enhanced degranulation and cytokine production in response to HSV1716-infected target cells, independent of a specific surface receptor.

### 3.3 HSV1716 infects NK cells

**Figure 3:**
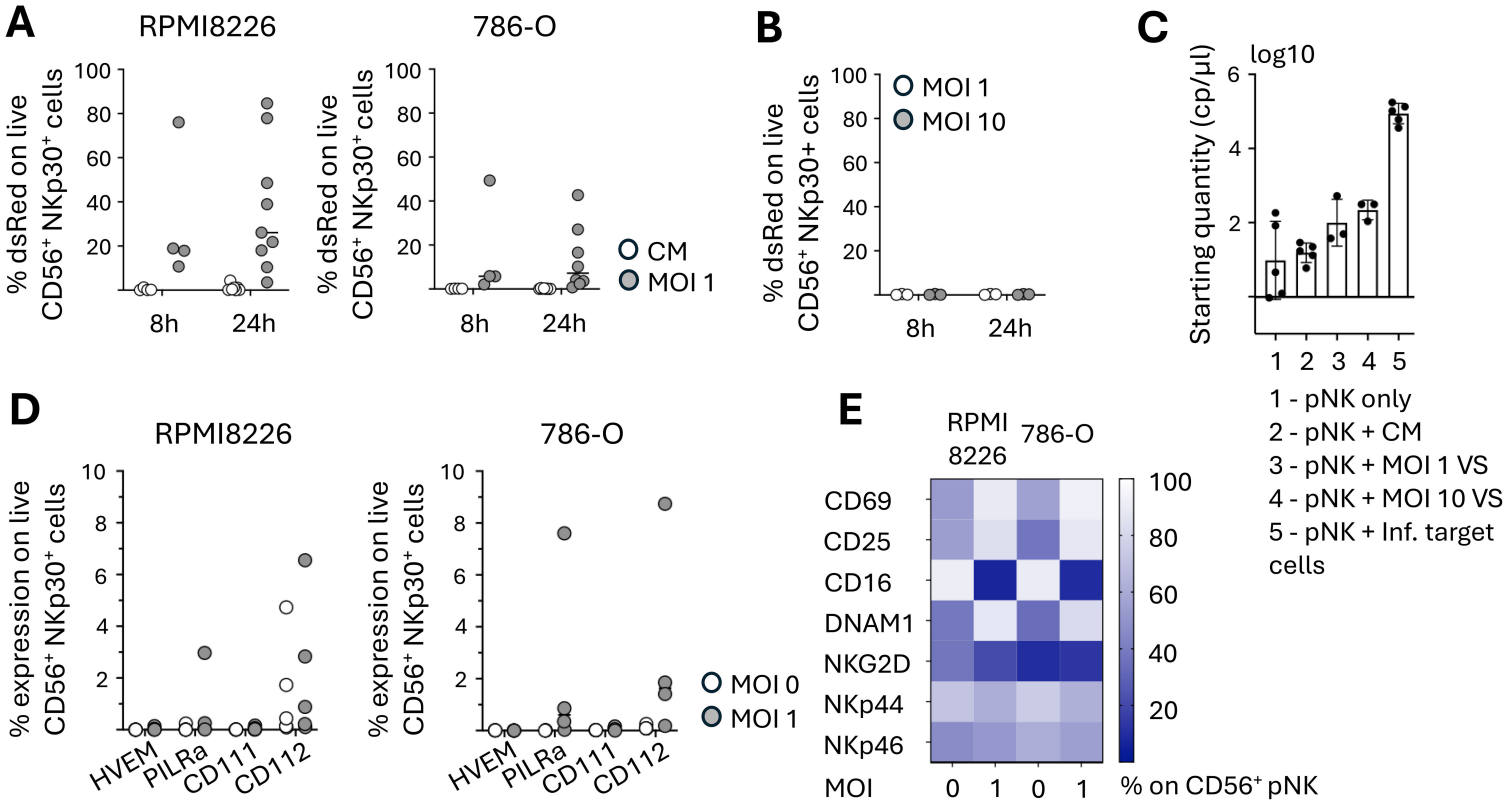
HSV1716 infects NK cells. **(A)** NK cells were co-cultured with HSV1716-infected RPMI8226 or 786-O target cells, and dsRed expression in NK cells was quantified at 8 h and 24 h post-infection. **(B)** NK cells were directly infected with HSV1716 at a multiplicity of infection (MOI) of 1 or 10, and dsRed expression was assessed at 8 h and 24 h post-infection. **(C)** Quantitative PCR (qPCR) was performed to measure HSV1716 viral DNA copy numbers in NK cells. **(D)** Cell surface expression of known HSV1 entry receptors was analyzed on NK cells cultured with HSV1716-infected (MOI 1) and mock-infected (MOI 0) target cells. Each dot represents the mean value from one donor, with experiments performed in duplicate. **(E)** Expression of NK cell activation markers and activating receptors was assessed in HSV1716-infected and mock-infected NK cells. Four donors were analyzed, with experiments performed in duplicate.

We hypothesized that viral interaction might contribute to the receptor-independent activation of NK cells. To investigate this, we monitored the expression of virally encoded dsRed on NK cells after 8 and 24 hours of co-culture with either infected target cells or conditioned medium (CM) (**Figure 3A**). NK cells expressed dsRed as early as 8 hours post-co-culture with HSV1716-infected targets. Conversely, NK cells cultured in CM or directly inoculated with the virus (MOI 1 or 10) showed no dsRed expression (**Figure 3B**). Quantitative PCR confirmed that HSV1716 infects NK cells (**Figure 3C**; **Supplementary Figure 1**). Although low viral titers were detected in NK cells exposed to CM or viral supernatant, the highest viral loads were consistently measured in NK cells co-cultured with infected targets. These findings suggest that, while HSV1716 can be acquired by NK cells in the absence of target-cell contact, direct cell-to-cell interactions substantially enhance viral load. Analysis of HSV entry receptors revealed that while HVEM remained undetectable, co-culture with infected targets slightly increased the expression of PILR*α* and CD112 on NK cells (**Figure 3D**). Finally, infected NK cells exhibited an activated phenotype, characterized by the upregulation of CD69, CD25, and DNAM1, and the downregulation of CD16 (**Figure 3E**). Given the lytic activity of HSV1716 on tumor target cells, we assessed NK cell viability following co-culture with infected or mock-infected tumor cells (**Supplementary Figure 2**). We observed a more rapid decline in NK cell viability during the first 24 hours of co-culture with infected target cells compared to non-infected controls, which subsequently stabilized. This pattern suggests that HSV1716 infection promotes NK cell lysis, with a small fraction of cells remaining viable after 48 and 72 hours. Collectively, these findings indicate that HSV1716 efficiently infects NK cells, leading to an activated phenotype while simultaneously promoting lysis in a subset of NK cells.

### 3.4 HSV1716 infection impairs the tumor-killing capacity of NK cells

**Figure 4:**
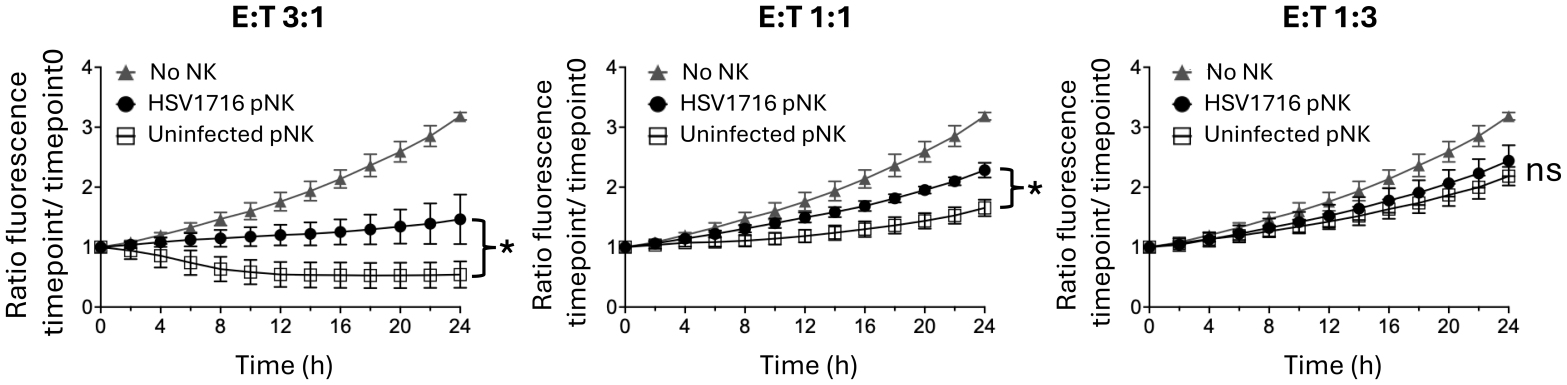
HSV1716 infected NK cells show impaired capacity to kill tumor target cells. **(A)** NK cells from healthy donors were co-cultured for 24 hours with HSV1716-infected 786-O cells. After isolation, the NK cells were transferred to uninfected 786-O target cells expressing the red fluorescent protein mKate2 at different effector to target ratios (E:T). Fluorescence was monitored in the Incucyte LiveCell Imager for an additional 24 hours. Fluorescence intensity at each time point was normalized to the value at time 0, with decreasing fluorescence indicating target-cell killing. Each dot represents the mean of six donors, analyzed in triplicates across three independent experiments. Statistical differences were assessed using two-way ANOVA. *p=0.05; ns, nonsignificant.

We next assessed the functional capacity of infected NK cells to kill uninfected tumor cells. Killing activity was evaluated using IncuCyte live-cell imaging by monitoring the loss of red fluorescent protein mKate2 (**Figure 4**). We found that infected NK cells were significantly less effective at killing compared to uninfected controls. This functional impairment was most pronounced and statistically significant at higher E:T ratios, while differences at lower ratios did not reach significance. Interestingly, preliminary observations indicated the appearance of a red fluorescent signal in initially uninfected tumor target cells after approximately 48 hours of co-culture with HSV1716-infected NK cells, suggesting a potential transfer of viral material from infected NK cells to uninfected tumor cells (**Supplementary Figure 3**). These findings raise the possibility that HSV1716 may be re-transmitted to tumor target cells through interactions with infected NK cells.

### 3.5 HSV1716 infection drives NK cells into a distinct transcriptional state with reduced effector gene expression

**Figure 5:**
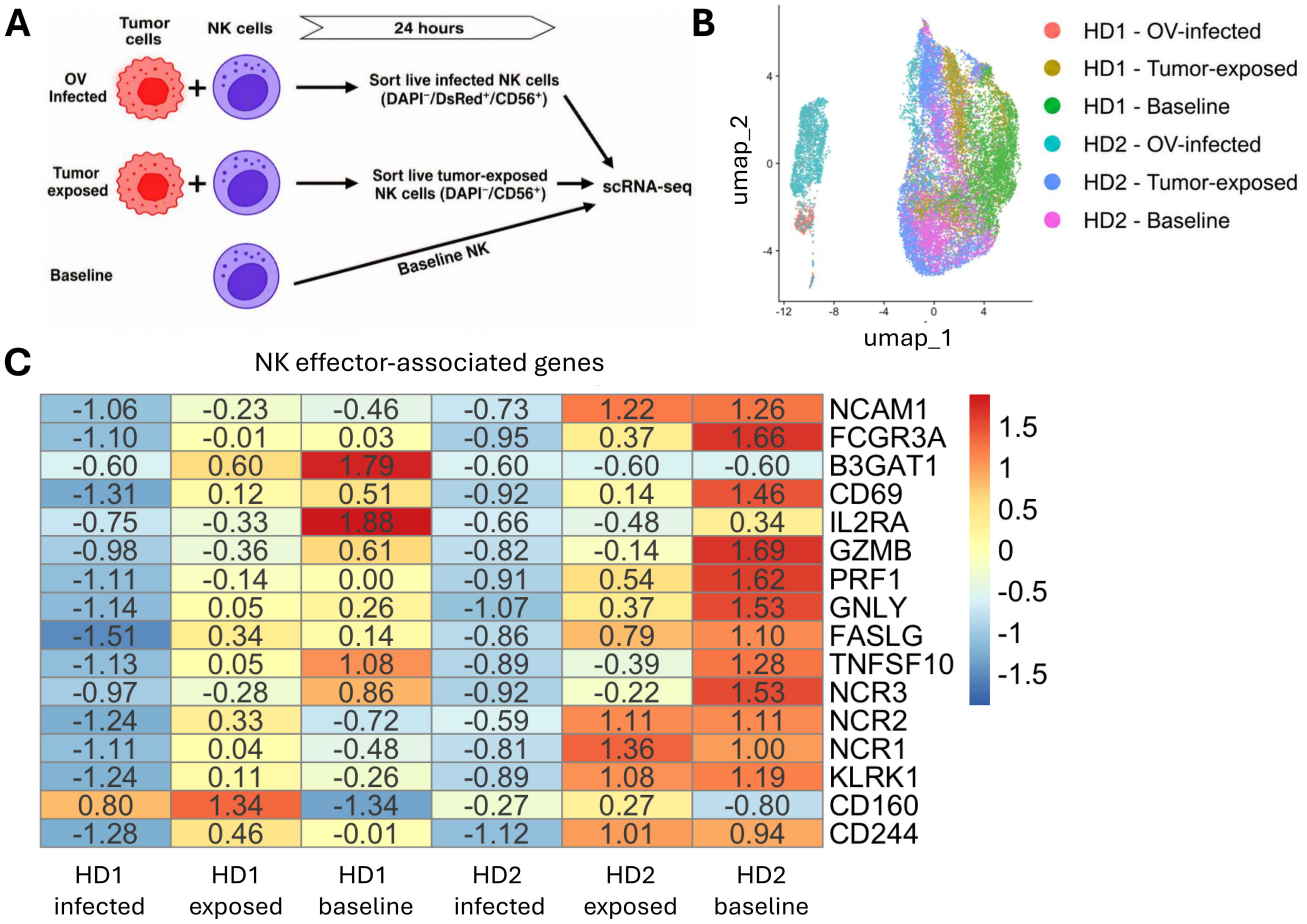
HSV1716 infected NK cells display a distinct transcriptional state associated with reduced expression of effector-associated genes. **(A)** Schematic representation of experimental workflow. **(B)** UMAP representation of integrated scRNA-seq data from HSV1716-infected, tumor-exposed, and baseline NK cells derived from two healthy donors (HD). **(C)** Heatmap showing average normalized expression of selected NK effector-associated genes.

We were further interested in the transcriptional profile of infected NK cells and performed single cell RNA sequencing (**Figures 5A–C**; **Supplementary Figures 4**-**6**). HSV1716-infected NK cells displayed lower overall recovery compared to control populations but retained comparable QC profiles after filtering, supporting their inclusion in downstream transcriptomic analyses (**Supplementary Figure 4**). UMAP projection revealed that HSV1716-infected NK cells formed a transcriptionally distinct cluster separated from tumor-exposed and baseline NK cells across both donors, indicating substantial transcriptional remodeling induced by HSV1716 infection. In contrast, tumor exposure alone was not sufficient to induce major transcriptional reprogramming, as tumor-exposed and baseline NK cells displayed largely overlapping distributions (**Figure 5B**). Infected NK cells displayed reduced expression of canonical NK effector-associated genes, including GZMB, PRF1, GNLY, NCR1, and NCR3, compared with tumor-exposed and baseline NK cells (**Figure 5C**). Tumor-exposed NK cells displayed an intermediate transcriptional phenotype, whereas baseline NK cells retained a strong mature cytotoxic transcriptional signature, with some donor-dependent variation observed (**Figure 5C**). A detailed description of the selected NK effector-associated genes is provided in **Supplementary Table 2** (**Supplementary Table 2**). To assess whether the observed transcriptional changes were attributable to reduced cell viability rather than infection, we evaluated mitochondrial RNA content and the expression of apoptosis- and cell death-related genes (**Supplementary Figures 4** and **5A**). Following quality control, infected samples consistently exhibited lower mitochondrial RNA percentages than their corresponding non-infected controls across both donors, indicating that infected cells did not show evidence of increased cell stress or death. Consistent with this, expression of the pro-apoptotic genes BAX, BBC3, and APAF1 was reduced in infected samples, while no upregulation of key apoptosis and death receptor signaling genes (CASP3, CASP8, FAS, and TNFSF10) was observed. In contrast, the stress-response genes HSPA1 and HSPA2 were upregulated in infected cells. Together, these findings suggest that the transcriptional alterations detected in infected cells are unlikely to be explained by increased apoptosis or loss of cell viability. Additional activation-associated transcriptional signatures were also assessed (**Supplementary Figure 5B**). HSV1716-infected NK cells displayed decreased expression of activation-associated genes, whereas tumor-exposed NK cells generally showed an intermediate phenotype. KEGG pathway enrichment analysis of upregulated differentially expressed genes (DGEs) revealed significant enrichment of stress-response pathways, including the MAPK, p53, and FoxO signaling pathways (**Supplementary Figure 6A**). In contrast, downregulated DGEs were significantly enriched for pathways related to cell adhesion, NK cell-mediated cytotoxicity, and other infection-associated pathways (**Supplementary Figure 6B**). Together, these findings indicate that HSV1716 infection promotes a stress-associated transcriptional program while suppressing the expression of genes involved in NK-cell effector functions, suggesting functional reprogramming of infected NK cells.

## 4 Discussion

This study investigates the interaction between a clinically evaluated HSV-based OV and primary human NK cells, two modalities of increasing importance in cancer immunotherapy, particularly in hematological malignancies. Despite their growing clinical relevance, the interplay between OV and NK cells remains insufficiently understood. Here, we demonstrate that NK cells can be directly infected by an HSV-based OV, HSV1716, resulting in reduced cytotoxic capacity and impaired tumor cell killing *in vitro*. Collectively, our results provide a novel perspective on NK cell biology and highlight an additional layer of complexity in NK-OV interactions. As both therapeutic platforms continue to advance in preclinical and clinical settings, a deeper understanding of these dynamics may be important for refining combinatorial strategies and overcoming current translational challenges.

HSV1-based vectors are among the most extensively explored platforms for oncolytic virotherapy due to their large genome and established manufacturing pipelines; however, HSV1 also encodes multiple immune-evasion mechanisms that alter NK-cell recognition of infected cells (11). A conserved strategy among herpesviruses is the modulation of ligands for activating NK-cell receptors, particularly NKG2D ligands, which are frequently retained intracellularly or downregulated from the cell surface following HSV1 infection (12, 13). Consistent with these observations, we found that HSV1716 infection reduced surface expression of MICA/B and several ULBP family members across tumor cell lines, supporting the notion that oncolytic HSV1 retains key features of wild-type HSV1 NK immune evasion. In contrast, modulation of DNAM1 ligands appears to be more context-dependent. While CMV broadly downregulates both CD112 and CD155 through UL141-mediated mechanisms (14, 15), HSV1 has been reported to incompletely affect these ligands, in line with the lower affinity of HSV1 glycoprotein D for CD112 compared with HSV2 (16, 17). In our study, HSV1716 induced cell-line-specific changes in CD112 and CD155 expression, with increased CD112 on multiple myeloma cell lines but reduced expression on selected solid tumor lines. Given that CD112 (nectin-2) functions both as a DNAM-1/TIGIT ligand and as an HSV1 entry receptor (18), its upregulation may reflect preferential infection or selective survival of susceptible tumor cell subsets. Finally, HSV1 has been shown to induce ligands for NCR through expression of the immediate-early protein ICP0, thereby promoting NK-cell activation despite preserved MHC class I expression (7, 19). In line, we observed enhanced NK-cell degranulation despite limited loss of HLA-A/B/C on HSV1716-infected targets. Together, these data indicate that HSV1716 infection induces a complex and dynamic remodeling of NK-cell–relevant ligands, integrating activating and inhibitory signals that likely vary with cell type and infection stage.

NK cells are generally regarded as key antiviral and antitumor effector cells in the context of oncolytic virotherapy. Multiple studies have reported enhanced NK-cell activation and degranulation towards tumor cells infected with HSV-based OV (20, 21). Consistent with these observations, our data confirm robust NK-cell activation in response to HSV1716-infected target cells. However, extending beyond previous work, our study reveals distinct functional alterations in NK cells following HSV1716 infection. To our knowledge, this is the first study demonstrating that an HSV-based OV can infect NK cells and substantially impair their killing capacity, uncovering a previously unrecognized level of complexity in NK–OV interactions. Campbell et al. previously showed that wild-type HSV1 and the related alphaherpesvirus varicella zoster virus (VZV) can enter primary human NK cells via a cell-associated mechanism and induce profound functional paralysis, characterized by reduced degranulation and cytokine production (22, 23). Our findings build on this work by demonstrating that an HSV1-based OV can infect NK cells. Notably, in contrast to Campbell et al., HSV1716-infected NK cells exhibited increased surface expression of activation markers despite reduced transcription of activation-associated genes, yet still displayed impaired tumor cell killing, consistent with observations from wild-type herpesvirus infections. These findings challenge the conventional view of NK cells as solely antiviral and antitumor effectors and instead point toward a more complex interaction, in which viral exposure can simultaneously drive activation while compromising functional competence. Our scRNA-seq analysis revealed that infected NK cells exhibited an increased stress response together with reduced expression of NK cell effector programs. Importantly, the observed transcriptional reprogramming is unlikely to be driven primarily by loss of cell viability as infected samples did not show increased mitochondrial RNA content, pro-apoptotic genes were generally downregulated, and genes involved in death receptor signaling were not induced. Instead, these alterations are likely the result of multiple host- and virus-mediated mechanisms. However, the descriptive nature of the present study limits mechanistic interpretation. Further investigations using targeted experimental approaches, including gene-deleted viral mutants, will be necessary to define the viral and host factors contributing to this phenotype. Finally, the translational significance of these *in vitro* findings remains to be determined and will require validation in future studies. Notably, detailed investigations of HSV1 infection in primary human NK cells *in vivo* are currently lacking, limiting assessment of the clinical relevance of our observations and underscoring the need for further studies to better understand the biological consequences of NK-cell infection in a therapeutic setting.

Based on our and previous data, we propose that direct cell-to-cell transfer of HSV1716 to NK cells may occur; however, the underlying mechanism remains unclear. Although HSV1 entry via viral glycoproteins and well-characterized cellular entry receptors represents the most extensively studied route, additional mechanisms have been described. These include direct cell-to-cell spread mediated by adhesion molecules as well as receptor-independent uptake through endocytic processes (24). In our study, expression of the classical HSV entry receptors remained low in both uninfected and infected NK cells. HSV1716 copy numbers increased in NK cells following direct contact with infected target cells, consistent with previous observations suggesting that cell-to-cell interactions facilitate viral transfer or viral load in immune cells (22, 23). The precise molecular mechanisms underlying this phenomenon remain unclear. NK-cell activation upon target-cell recognition may create a cellular environment that is more permissive to viral transfer as well as viral gene expression and replication through enhanced transcriptional and metabolic activity. This possibility is supported by our qPCR data, which show low levels of viral DNA in NK cells directly exposed to viral inoculum, suggesting that viral uptake can occur independently of target-cell contact, but total viral load is significantly increased in NK cells exposed to infected target cells. Data regarding HSV1 infection of primary human immune cells, particularly NK cells, remain limited, and the mechanisms governing viral entry and persistence in these cell types are incompletely understood. Previous work by Aubert et al. demonstrated that HSV can infect both CD4^+^ and CD8^+^ T cells, isolated from human herpetic lesions (25). Viral transfer occurred far more efficiently through direct cell-to-cell contact than through exposure to cell-free virus. Importantly, T-cell activation enhanced susceptibility to infection, and viral transfer was mediated through an LFA-1-dependent virological synapse-like structure. Future studies employing receptor-blocking approaches, genetic ablation of candidate entry factors, or experimental modulation of NK-cell activation and target-cell contact will be necessary to dissect the relative contribution of these mechanisms. Importantly, these findings should be interpreted in the context of their clinical relevance. As most oncolytic viruses are administered intratumorally, infection of tumor-infiltrating immune cells is biologically plausible. However, the clinical significance of such interactions remains unclear. Although oncolytic virotherapy is generally considered safe and feasible, our findings suggest that interactions between oncolytic viruses, immune cells, and tumor cells may influence therapeutic activity and therefore warrant further investigation. Consequently, the biological and translational relevance of these observations will require validation in more physiologically relevant settings, including *in vivo* models and analyses of patient-derived samples.

The ability of HSV1 and its oncolytic derivatives to infect NK cells is intriguing from several perspectives. Although early clinical studies investigating either systemic or local administration of HSV1716 reported no detectable viral shedding or viremia, HSV1–derived viral sequences were detected by PCR in peripheral blood samples from a subset of treated patients (26, 27). Notably, viral DNA could be detected for up to 28 days following both local and systemic administration. However, the specific cell populations harboring HSV1716 were not identified, and the clinical relevance of these findings - either with respect to therapeutic efficacy or adverse events - remains unclear. To date, oncolytic virotherapy has generally demonstrated a favorable safety profile and is widely considered to be a safe treatment modality. In our study, *in vitro* tumor cell killing at lower effector-to-target ratios was not significantly altered, suggesting that the *in vivo* relevance of HSV1716 infection of NK cells for oncolytic virotherapy or combination strategies cannot yet be fully determined. Nevertheless, our findings raise the possibility that NK cells might act as carrier cells for HSV-based OV. In line with this concept, previous studies have demonstrated that HSV1716 can directly infect macrophages (28). Importantly, HSV1716-infected macrophages have been successfully employed as carrier cells in murine models of triple-negative breast cancer metastasis, resulting in improved tumor control compared to systemic viral delivery while requiring lower viral doses. Carrier-cell–based delivery approaches for oncolytic viruses are currently under active development, and early-phase clinical studies have begun to explore their feasibility (29). Such strategies are particularly relevant given that systemically administered OV are rapidly neutralized by pre-existing antibodies. This limitation is especially pronounced for HSV-based OV, as HSV1 seroprevalence reaches up to 70% in the adult population (30), significantly constraining systemic delivery approaches. Our data demonstrate that NK cells - already under clinical investigation as cell-based cancer immunotherapies - can be loaded with HSV1716. The preliminary data presented here raise the possibility that infected NK cells may transfer virus to tumor target cells. However, these findings should be interpreted with caution given the exclusively *in vitro* nature of the study. Further investigations in physiologically relevant *in vivo* models are required to determine whether such mechanisms occur *in vivo* and contribute to therapeutic outcome. Nevertheless, these observations warrant further exploration of the feasibility and potential therapeutic utility of NK cells as carrier cells for HSV-based oncolytic virotherapy.

Overall, OV offer a versatile platform with considerable promise as anticancer immunotherapies. However, multiple biological and translational challenges remain that currently limit their broader clinical application. A deeper understanding of the complex interactions between OV and the immune system will be essential for the rational optimization of OV-based therapeutic strategies.

## Data Availability

The raw sequencing data have been deposited in the Gene Expression Omnibus (GEO) and are available under accession number GSE338854 (https://www.ncbi.nlm.nih.gov/geo/query/acc.cgi?acc=GSE338854).

## Supplementary material

Supplementary Figure 1 NK cell purity control. Primary human NK cells were isolated from healthy donor buffy coats by density gradient centrifugation (Lymphoprep) followed by negative magnetic bead selection prior to qPCR. **(A)** Flow cytometry gating strategy used to determine the proportions of CD3^+^ T cells and CD56^+^ NK cells. **(B)** Frequencies of NK cells and T cells before and after NK cell isolation. Each dot represents one donor.

Supplementary Figure 2 HSV1716 enhances NK cell death. Primary human NK cells were co-cultured with HSV1716 infected (MOI 1) or mock-infected (MOI 0) tumor target cells and cell viability of CD56^+^ NKp30^+^ NK cells was measured 24h, 48h and 72h after co-culture start.

Supplementary Figure 3 dsRed expression in uninfected target cells following co-culture with HSV1716-infected pNK cells. Representative fluorescence microscopy images showing dsRed expression at the start of co-culture and after 48 hours of co-culture between HSV1716-dsRed-infected pNK cells and uninfected 786-O wild-type target cells. Red arrows indicate dsRed fluorescence detected in target cells, suggesting transfer or spread of viral infection from infected pNK cells to initially uninfected 786-O cells.

Supplementary Figure 4 Quality control metrics and cell recovery across all scRNA-seq samples. **(A)** Pre-quality control (Pre-QC) distributions of detected genes per cell (nFeature_RNA), total transcript counts per cell (nCount_RNA), percentage of mitochondrial transcripts (percent.mt), and percentage of ribosomal transcripts (percent.ribo) across all samples prior to filtering. **(B)** Post-quality control (Post-QC) distributions of the same metrics following filtering and downstream processing. **(C, D)** Cell recovery before and after QC filtering for all samples derived from healthy donor 1 (HD1) and healthy donor 2 (HD2), respectively. HSV1716-infected NK cell populations were generated in two technical replicates per donor. Tumor-exposed NK cells correspond to NK cells co-cultured with non-infected tumor cells, while baseline NK cells were cultured in the absence of tumor cells. **(E)** Sample annotation and corresponding numerical identifiers used throughout the scRNA-seq analyses.

Supplementary Figure 5 HSV1716 infects NK cells. Heatmap showing average normalized expression of selected apoptosis **(A)** and activation **(A)** genes.

Supplementary Figure 6 Enrichment of up- and downregulated DGE. DGEs were identified by comparing infected co-culture NK cells with noninfected co-culture NK cells. **(A)** Pathway enrichment analysis of the five most upregulated DGEs using KEGG revealed significant enrichment of the MAPK, P53 and FoxO signaling pathways. **(B)** Pathway enrichment analysis of five most downregulated DGEs using KEGG revealed significant enrichment of pathways involved in cell adhesion, NK cell mediated cytotoxicity and infection related pathways.
