# Supplementary material for: Oncolytic HSV1716 attenuates tumor-killing activity of infected primary NK cells
Source: Front Immunol. 2026 Aug 3;17:1895832. doi: 10.3389/fimmu.2026.1895832 (PMC13478253; doi:10.3389/fimmu.2026.1895832)
Supplement: Supplementary file 1 [file Table1.docx]

**Supplementary Table 1**

| **Antigen** | **Clone** | **Fluorophore** | **Company** |
| --- | --- | --- | --- |
| gE | 9H3 | Conjugated with PE/ FITC or APC Lightning Link | abcam/VWR |
| HLA-A,B,C | W6/32 | PerCP-Cy5.5 | BioLegend |
| CD111 | CK41 | BV421 | BD Bioscience |
| CD112 | TX31 | PE-Cy7 | BioLegend |
| CD155 | TX24 | BV605 | BD Bioscience |
| MICA/B | 6D4 | BV711 | BD Bioscience |
| ULBP2,5,6 | 165903 | Conjugated with APC Lightning Link | R&D |
| CD56 | NCAM16.2 | BV605 | BD Bioscience |
| NKp30 | p30-15 | Alexa 647 | BioLegend |
| CD16 | 3G8 | BUV737 | BD Bioscience |
| KIR | EB6 (HP-MA4), GL183, and DX9 | Conjugated with PE Lightning Link | Invitrogen / Biotechne |
| NKp44 | p44-8 | BB515 | BD Bioscience |
| NKp46 | 9E2/Nkp46 | PE-Cy7 | BioLegend |
| DNAM1 | DX11 | BUV395 | BD Bioscience |
| NKG2C | FAB138N | Alexa 647 | R&D |
| NKG2D | 1D11 | BV785 | BioLegend |
| NKG2A | 131411 | BB700 | BD Bioscience |
| PD1 | EH12.1 | BV421 | BD Bioscience |
| LAG3 | 11C3C65 | PE-CF594 | BioLegend |
| TIGIT | 741182 | BUV615 | BD Bioscience |
| TIM3 | 7D3 | BV711 | BD Bioscience |
| CD69 | FN50 | BV421 | BD Bioscience |
| CD25 | M-A251 | PE-CF594 | BD Bioscience |
| CD270 (HVEM) | CW10 | BUV395 | BD Bioscience |
| PILR-α | Polyclonal | APC | R&D systems |

**Supplementary Table 2**
